# A Leucemia Mieloide Aguda Poderia ter se Apresentado Ainda Pior? “Causa Incomum de Trombose Multiarterial Concomitante”

**DOI:** 10.36660/abc.20220808

**Published:** 2023-07-21

**Authors:** Kutluhan Eren Hazir, Ersin Cagri Simsek, Esra Baldan, Hakan Gökalp Uzun, Hale Bulbul, Bengisu Yarci, Elif Busra Ozcan

**Affiliations:** 1 Izmir Tepecik Training and Research Hospital Izmir Turquia Izmir Tepecik Training and Research Hospital – Cardiology, Izmir – Turquia; 2 Izmir Tepecik Training and Research Hospital Izmir Turquia Izmir Tepecik Training and Research Hospital – Internal Medicine, Izmir – Turquia; 3 Izmir Tepecik Training and Research Hospital Izmir Turquia Izmir Tepecik Training and Research Hospital – Hematology, Izmir – Turquia; 4 Izmir Bozyaka Training and Research Hospital Izmir Turquia Izmir Bozyaka Training and Research Hospital – Neurology, Izmir – Turquia

**Keywords:** Leucemia Promielocítica Aguda, Infarto do Miocárdio, Infarto Cerebral

## Abstract

A leucemia promielocítica aguda (LPA) é um subgrupo da leucemia mieloide aguda (LMA). Embora se saiba que as complicações hemorrágicas são comuns, as complicações trombóticas não são tão raras quanto se pensa. No entanto, infarto do miocárdio e incidência de acidente vascular cerebral isquêmico são muito raros durante a LMA. Aqui, apresentamos o caso surpreendente de LPA diagnosticada com pancitopenia em sua apresentação com infarto agudo do miocárdio e acidente vascular cerebral isquêmico.

## Introdução

A leucemia promielocítica aguda (LPA ou LMA-M3) é um subgrupo de leucemia mieloide com um curso clínico e fisiopatologia diferente de outras formas de leucemia mieloide aguda (LMA).^1^ É bem conhecido que a coagulação intravascular disseminada (CID) e a leucocitose que se desenvolvem durante a LMA podem causar hipercoagulopatia.^2^ Sabe-se também que a frequência de eventos cardiovasculares aumenta durante as doenças malignas; no entanto, infarto do miocárdio (IM) e acidente vascular cerebral isquêmico são muito raros no curso da LMA.^3^

## Relato de Caso

**Figura 1:**
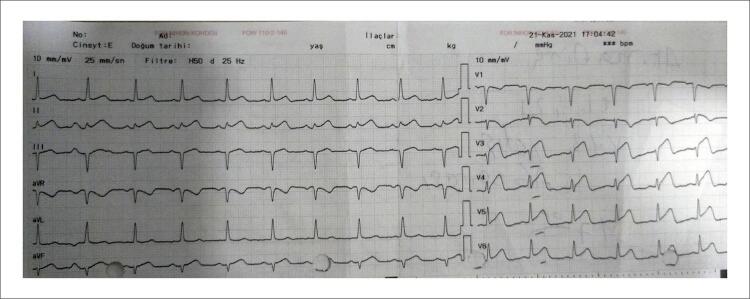
– Supradesnivelamento do segmento ST observado em D1, D2, V3, V4, V5, V6 no momento da admissão.

**Figura 2:**
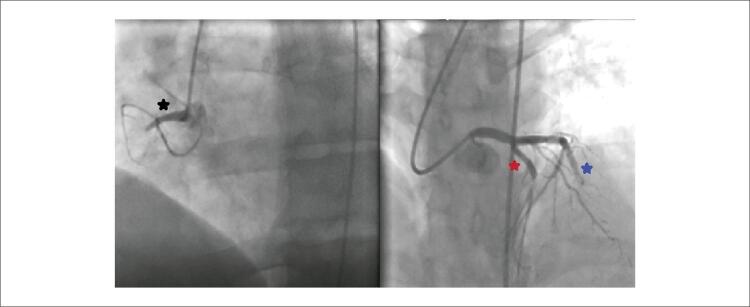
– A angiografia coronária mostra 100% de lesões trombosadas nas artérias artéria coronária direita, descendente anterior esquerda e circunflexa.

**Figura 3:**
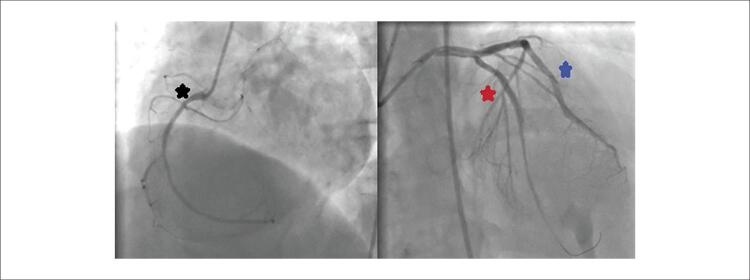
– Descendente anterior esquerda (DAE), circunflexa (Cx) e artéria coronária direita revascularizadas.

Paciente do sexo masculino, 49 anos, com história médica de hipertensão e doença arterial coronariana, deu entrada no pronto-socorro com quadro de angina. Dois anos antes da admissão, foi realizado implante de Stent na descendente anterior esquerda (DAE), circunflexa (Cx) e artéria coronária direita (CD) devido à síndrome coronariana aguda. Desde então, o paciente faz uso de ácido acetilsalicílico 100 mg uma vez ao dia, perindopril 5 mg e nebivolol 5 mg uma vez ao dia para hipertensão, que estava bem controlada no momento da admissão. O paciente foi encaminhado ao laboratório de cateterismo cardíaco após constatação de supradesnivelamento do segmento ST nas derivações anteriores ao eletrocardiograma ( Figura 1 ). Uma coronariografia de emergência revelou oclusões totais nas três principais artérias coronárias ( Figura 2 ). Os stents DAE, OM1 e CD foram feitos com sucesso com stent farmacológico (SF) 3,02x24mm, SF 2,75x31mm e SF 2,75x16mm. O fluxo TIMI 3 foi fornecido em todos os 3 vasos. Após revascularização ad hoc de 3 vasos com sucesso ( Figura 3 ), o paciente iniciou ácido acetilsalicílico 100 mg, clopidogrel 75 mg, atorvastatina 80 mg, metoprolol 50 mg, ramipril 2,5 mg e pantoprazol 40 mg e foi acompanhado na unidade de terapia intensiva coronariana.

**Figura 4:**
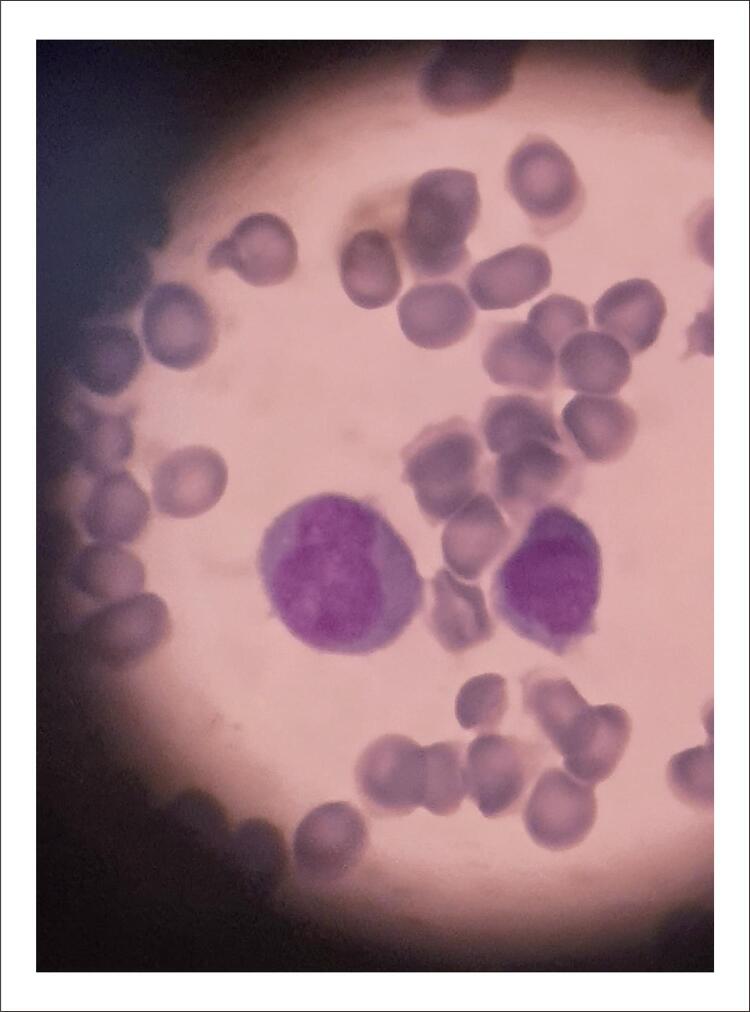
– Blastos finamente granulados com núcleos bilobados observados ao microscópio em esfregaços de aspirado de medula óssea.

A ecocardiografia transtorácica revelou que a fração de ejeção do ventrículo esquerdo (FEVE) era de 30%. Havia aneurismas e hipocinesia grave no ápice, hipocinesia grave na parede anterior e hipocinesia leve nas paredes inferior e lateral. Não foi observado trombo intracardíaco. Testes laboratoriais revelaram que a contagem de glóbulos brancos (CGB) era de 0,6 109/L, a hemoglobina era de 6,5 g/dL, a contagem de plaquetas era de 61109/L, o dímero D era de 3 mg/L e o fibrinogênio (FIB) era de 2,69 g/L.TO paciente apresentava pancitopenia nos exames de sangue realizados após revascularização de emergência sem hepatoesplenomegalia e linfadenopatia ao exame físico. Petéquias, púrpura e equimoses não foram observadas. A fim de determinar a etiologia subjacente da pancitopenia grave observada no hemograma completo do paciente, uma biópsia de medula óssea foi realizada. Os esfregaços do aspirado e da biópsia de medula óssea mostraram uma medula hipercelular com infiltração difusa de blastos finamente granulados cujos núcleos eram frequentemente bilobados com contorno dobrado ( Figura 4 ). O tempo de protrombina (TP), tempo de tromboplastina parcial ativada (aPPT) e FIB estavam todos dentro da faixa normal.

**Figura 5:**
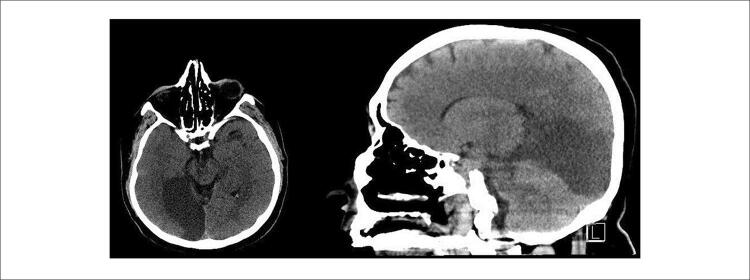
– Infarto subagudo em occipital direito e lobo temporal posteromedial direito visto na tomografia computadorizada do paciente.

O paciente relatou perda de visão no segundo dia de acompanhamento na unidade de terapia intensiva. Apenas hemianopsia homônima esquerda foi descoberta no exame neurológico como achado patológico. A imagem neurocraniana revelou um infarto subagudo nas regiões occipital direita e lobo temporal posteromedial direito compatível com a área de suprimento da artéria cerebral posterior (ACP) ( Figura 5 ). A tomografia computadorizada de cabeça e pescoço com contraste revelou que as artérias carótidas comuns (ACC), carótidas internas (ACI) e carótidas externas (ACE) bilaterais eram normais. Não havia estenose significativa ou dilatação aneurismática.

A terapia antiplaquetária (ácido acetilsalicílico e clopidogrel) que o paciente vinha fazendo foi mantida. Em um exame mais aprofundado do paciente com múltiplos eventos trombóticos, o *anticorpo antinuclear* (ANA) e o painel de trombofilia hereditária foram negativos. A translocação cromossômica t (15;17) foi detectada. O estudo de citometria de fluxo do aspirado de medula óssea revelou 62% de blastos com CD117, CD33 e forte positividade para CD34 e imunofenótipo HLA-DR negativo. À luz desses achados clínicos, o paciente foi diagnosticado com a variante microgranular de (LPA).

Durante a preparação para a quimioterapia, o paciente apresentou colapso hemodinâmico súbito. Uma patologia cardíaca aguda não foi considerada porque não foram observadas alterações isquêmicas ativas no ECG. Devido à insuficiência respiratória e hipotensão, o paciente morreu em um curto período. A autópsia não pôde ser realizada no caso. A angiografia neurocraniana e da artéria pulmonar não pôde ser realizada devido aos esforços de ressuscitação cardiopulmonar. No entanto, a parada respiratória pode ter se desenvolvido devido à hemorragia pontina no paciente pancitopênico recebendo terapia antiplaquetária terapia ou uma embolia pulmonar recém-desenvolvida, além de eventos trombóticos coronarianos. Além disso, no paciente com FEVE baixa, a causa do colapso hemodinâmico súbito pode estar relacionada ao choque cardiogênico.

## Discussão

Neste caso, apresentamos um caso recém-diagnosticado de LMA-variante microgranular com múltiplas tromboses catastróficas nas artérias coronárias e complicadas com acidente vascular cerebral no momento do diagnóstico.

A variante microgranular é responsável por cerca de 25% de todos os casos de LMA. A variante microgranular da LPA foi considerada em nosso paciente por causa dos blastos finamente granulados com núcleos bilobados na avaliação morfológica. Ao contrário da variante hipergranular, a positividade de CD34 é mais comum na variante microgranular. Da mesma forma, a forte positividade de CD34 em nosso caso apoiou o diagnóstico de LPA microgranular. O CD34 fraco/ausente é patognomônico para o diagnóstico de LPA. No entanto, em casos raros, a LPA pode apresentar um forte imunofenótipo positivo para CD34.^4^ Por outro lado, em casos de forte positividade para CD34, os subtipos de LMA não-LPA devem ser considerados no diagnóstico diferencial. Em nosso paciente com múltiplas tromboses catastróficas, CID não foi considerado como FIB, aPTT, TP e INR foram todos considerados normais. Foi diagnosticado por fenotipagem morfológica e imunológica. Embora as complicações hemorrágicas e relacionadas à infecção sejam encontradas principalmente (10-20%) no curso clínico da LPA, as complicações trombóticas devido à CID também podem ser raramente observadas.^5 - 9^ O número de estudos sobre a incidência de desenvolvimento de trombose no momento do diagnóstico antes da terapia de indução é muito limitado.^7^

O infarto agudo do miocárdio (IAM) durante a LMA não é frequentemente encontrado na prática clínica. O IAM ocorre mais comumente devido à trombose aguda, que, juntamente com a ruptura ou erosão das placas desenvolvidas por um fundo aterosclerótico, obstrui as artérias coronárias secundárias à trombogênese local e sistêmica.^9^ Além disso, condições que causam desequilíbrio na oferta e na demanda de oxigênio pelo miocárdio também podem causar IAM não obstrutivo.^9 , 10^ Doenças malignas também são conhecidas por causar um aumento na frequência de IAM devido a vasoespasmo, trombose, aterosclerose acelerada e dano endotelial relacionado à radiação.^3 , 11^ Um relato de caso afirmou que IAMSST inferior se desenvolveu em um paciente LPA com CID, e a síndrome coronariana aguda regrediu dramaticamente em um curto período de tempo com infusão de heparina.^12^ Os autores consideraram que a etiologia da síndrome coronariana aguda do paciente foi devida à CID e não à ruptura da placa aterosclerótica.^12^ Casos de IAM não trombótico secundário a hiperleucocitose e leucostase e IAM trombótico secundário a CID têm sido relatados na literatura.^13 , 14^ Curiosamente, neste caso que apresentamos, não houve hiperleucocitose, leucostase ou CID. Sabe-se também que, principalmente na LPA, os promielócitos leucêmicos podem aumentar a expressão do fator tecidual (FT) e do pró-coagulante do câncer (PC), levando à trombose.^7^ O desenvolvimento de trombose existente sem coagulopatia pode ser considerado secundário ao conteúdo pró-coagulante nos grânulos de promielócitos anormais.

A coocorrência de doenças cerebrovasculares (DCV) com leucemia, que possuem fatores de risco semelhantes ao IAM, não é comum na comunidade. No entanto, um estudo mostrou que o risco de desenvolvimento de doença cerebrovascular é maior em pacientes com leucemia em comparação com a população normal. A taxa de mortalidade aumenta cinco vezes mais em pacientes com leucemia com desenvolvimento de DCV em comparação com pacientes com leucemia sem ela.^15 , 16^ As DCV isquêmicas são uma complicação extremamente rara na leucemia.^15^ Além disso, foi relatado um caso nos EUA em que um paciente apresentou trombose aguda de stent coronariano de extremidade inferior e tardia, que se pensava ser secundária a CID, e foi diagnosticado com LMA-M3 durante a hospitalização.^14^ Além disso, eventos tromboembólicos arteriais-venosos recorrentes com risco de vida (como trombose venosa profunda, embolia pulmonar, infarto esplênico, infarto renal, etc.) foram relatados em pacientes com LPA na literatura, principalmente durante a terapia de indução de quimioterapia.^17^

Xiao et al. relataram que CGB/dímero-D alta (>5) e FIB/dímero-D baixa (<5) poderiam ser preditores independentes para trombose em seu estudo em pacientes com LPA.^18^ No caso que apresentamos, a relação FIB/dímero-D foi baixa em correlação com este estudo (1,1), mas diferentemente, a relação CGB/dímero-D não foi alta (0,2). Essa diferença pode ser explicada pelo fato de nosso caso ser pancitopênico, enquanto pacientes com leucócitos muito altos foram incluídos no estudo de referência.

Pensamos que os múltiplos eventos trombóticos experimentados pelo paciente eram principalmente devidos à LMA causando coagulopatia secundária ao conteúdo pró-coagulante nos grânulos de promielócitos anormais. Ao mesmo tempo, aneurisma apical e hipocinesia grave no ápice também são possíveis na etiologia do AVC cardioembólico, apesar da ausência de trombo intracardíaco na ecocardiografia após IAM.

Neste relato de caso, apresentamos um paciente com IAM que desenvolveu *acidente vascular cerebral* durante o acompanhamento e foi diagnosticado com LMA-M3 com resultados catastróficos. Embora a LMA-M3 tenha o melhor prognóstico entre as leucemias mieloides, é óbvio que o prognóstico é prejudicado pela coexistência de IAM e acidente vascular cerebral. Embora a CID e a leucostase sejam as culpadas na maioria dos casos de IAM relacionados à LMA relatados na literatura, esses quadros clínicos não estavam presentes em nosso caso. A LPA deve ser considerada no diagnóstico diferencial de trombose acompanhada de pancitopenia.

Nos casos que causam isquemia multiarterial, as doenças hematológicas devem ser incluídas na investigação etiológica. Possíveis patologias devem ser avaliadas por estudo de hemograma completo, dímero D e FIB. O diagnóstico da doença primária subjacente é importante para determinar o método de tratamento (como medicação antitrombótica, leucaférese ou intervenções arteriais percutâneas) e o prognóstico.
