# Supplementary material for: A Leucemia Mieloide Aguda Poderia ter se Apresentado Ainda Pior? “Causa Incomum de Trombose Multiarterial Concomitante”
Source: Arq Bras Cardiol. 2023 Jul 21;120(8):20220808. [Article in Portuguese] doi: 10.36660/abc.20220808 (PMC10464858; doi:10.36660/abc.20220808)
Supplement: Translation

# Translation: Could Acute Myeloid Leukemia Have Presented Even Worse? “Uncommon Cause of Concurrently Multivessel Thrombosis”

**Authors:** Kutluhan Eren Hazir, Ersin Cagri Simsek, Esra Baldan, Hakan Gökalp Uzun, Hale Bulbul, Bengisu Yarci, Elif Busra Ozcan

**Affiliations:** 1 Turquia Izmir Tepecik Training and Research Hospital – Cardiology, Izmir – Turquia; 2 Turquia Izmir Tepecik Training and Research Hospital – Internal Medicine, Izmir – Turquia; 3 Turquia Izmir Tepecik Training and Research Hospital – Hematology, Izmir – Turquia; 4 Turquia Izmir Bozyaka Training and Research Hospital – Neurology, Izmir – Turquia

**Keywords:** Leukemia, Promyelocytic, Acute, Myocardial Infarction, Cerebral Infarction

## Abstract

Acute promyelocytic leukemia (APL) is a subgroup of acute myeloid leukemia (AML). Although it is known that hemorrhagic complications are common, thrombotic complications are not as rare as thought. However, myocardial infarction and ischemic stroke incidence are very rare during AML. Here, we present the astonishing case of APL diagnosed with pancytopenia in its presentation with acute myocardial infarction and ischemic stroke.

## Introduction

Acute promyelocytic leukemia (APL or AML-M3) is a subgroup of myeloid leukemia with a clinical course and pathophysiology different from other forms of acute myeloid leukemia (AML).^1^ It is well known that disseminated intravascular coagulation (DIC) and leukocytosis that develop during AML may cause hypercoagulopathy.^2^ It is also known that the frequency of cardiovascular events increases during malignant diseases; however, myocardial infarction (MI) and ischemic stroke are very rare in the course of AML.^3^

## Case Report

A 49-year-old male patient with a medical history of hypertension and coronary artery disease was admitted to the emergency department with angina. Two years before admission, stent implantation was performed on the left anterior descending (LAD), circumflex (Cx), and right coronary artery (RCA) due to acute coronary syndrome. Since then, the patient has been taking acetylsalicylic acid 100 mg once daily along with perindopril 5mg and nebivolol 5mg once daily for hypertension which was well controlled at the time of the admission. The patient was taken to the cardiac catheterization laboratory after ST-segment elevation in the anterior leads was observed on the electrocardiogram ( Figure 1 ). An emergency coronary angiography revealed total occlusions in the three main coronary arteries ( Figure 2 ). Stenting LAD, OM1, and RCA were done successfully with drug-eluting stent (DES) 3.02x24mm, DES 2.75x31mm, and DES 2.75x16mm. TIMI 3 flow was provided in all 3 vessels. After successful ad hoc 3-vessel revascularization ( Figure 3 ). The patient was started on acetylsalicylic acid 100 mg, clopidogrel 75 mg, atorvastatin 80 mg, metoprolol 50 mg, ramipril 2.5 mg and pantoprazole 40 mg and was followed up in the coronary intensive care unit.

**Figure 1:** – ST-segment elevation observed in D1, D2, V3, V4, V5, V6 at the time of admission.

**Figure 2:** – Coronary angiography shows 100% thrombosed lesions in the right coronary artery, left anterior descending and circumflex arteries.

**Figure 3:** – Revascularized left anterior descending, circumflex and right coronary artery.

Transthoracic echocardiography revealed that the left ventricular ejection fraction (LVEF) was 30%. There were aneurysms and severe hypokinesia at the apex, severe hypokinesia at the anterior wall, and mild hypokinesia at the inferior and lateral walls. No intracardiac thrombus was observed. Laboratory tests revealed that white blood cell count (WBC) was 0.6 10^9^ /L, hemoglobin was 6.5 g/dL, platelet count was 6110^9^ /L, D-dimer was 3mg/L, and fibrinogen (FIB) was 2.69 g/L. The patient had pancytopenia in the blood tests performed after emergency revascularization without hepatosplenomegaly and lymphadenopathy in the physical examination. Petechiae, purpura, and ecchymosis were not observed. In order to determine the underlying etiology of the severe pancytopenia observed in the patient’s complete blood count, a bone marrow biopsy was performed. The bone marrow aspirate and biopsy smears showed a hypercellular marrow with diffuse infiltration of finely granulated blasts whose nuclei were often bilobed with a folded contour ( Figure 4 ). Prothrombin time (PT), activated partial thromboplastin time (aPPT), and FIB were all within the normal range.

**Figure 4:** – Finely granulated blasts with bilobed nuclei observed under a microscope in the bone marrow aspirate smears.

The patient described vision loss on the second intensive care unit follow-up day. Only left homonymous hemianopsia was discovered in the neurological examination as a pathological finding. Neurocranial imaging revealed a subacute infarct in the right occipital and right posteromedial temporal lobe regions compatible with the posterior cerebral artery (PCA) supply area ( Figure 5 ). Contrast-enhanced computed tomography of the head and neck revealed that bilateral common carotid arteries (CCA), internal carotid arteries (ICA), and external carotid arteries (ECA) were normal. There was no significant stenosis or aneurysmal dilatation.

**Figure 5:** – Subacute infarct in the right occipital and right posteromedial temporal lobe seen in the CT scan of the patient.

The antiplatelet therapy (acetylsalicylic acid and clopidogrel) the patient had been taking was continued. In further examination of the patient with multiple thrombotic events, the *antinuclear antibody* (ANA) and hereditary thrombophilia panel were negative. Chromosomal translocation t(15;17) was detected. The flow cytometric study of the bone marrow aspirate revealed 62% of blasts with CD117, CD33, and strong CD34 positivity and an HLA-DR negative immunophenotype. In light of these clinical findings, the patient was diagnosed with the microgranular variant of (APL).

During preparation for chemotherapy, sudden hemodynamic collapse developed in the patient. An acute cardiac pathology was not considered because no active ischemic changes were observed in the ECG. Due to respiratory failure and hypotension, the patient died in a short period. An autopsy could not be performed on the case. Neurocranial and pulmonary artery angiographic imaging could not be performed due to cardiopulmonary resuscitation efforts. However, respiratory arrest may have developed due to pontine hemorrhage in the pancytopenic patient receiving antiplatelet therapy or a newly developed pulmonary embolism in addition to coronary thrombotic events. In addition, in the patient with low LVEF, the cause of sudden hemodynamic collapse may be related to cardiogenic shock.

## Discussion

In this case, we have presented a case of newly diagnosed AML-microgranular variant with multiple catastrophic thromboses in coronary arteries and complicated with stroke at the time of diagnosis.

The microgranular variant is responsible for about 25 percent of all AML cases. The microgranular variant of APL was considered in our patient because of the finely granulated blasts with bilobed nuclei in the morphological evaluation. Unlike the hypergranular variant, CD34 positivity is more common in the microgranular variant. Similarly, the strong CD34 positivity in our case supported the diagnosis of microgranular APL. The weak/absent CD34 is pathognomic for the diagnosis of APL. However, in rare cases, APL can present a strong CD34 positive immunophenotype.^4^ On the other hand, in cases of strong CD34 positivity, non-APL AML subtypes should be considered in the differential diagnosis. In our patient with multiple catastrophic thromboses, DIC was not considered as FIB, aPTT, PT, and INR were all found to be normal. It was diagnosed by morphological and immune phenotyping. While hemorrhagic and infection-related complications are mostly encountered (10-20%) in the clinical course of APL, thrombotic complications due to DIC can also be rarely seen.^5 - 9^ The number of studies on the incidence of thrombosis developing at the time of diagnosis before induction therapy is very limited.^7^

Acute myocardial infarction (AMI) during AML is not frequently encountered in clinical practice. AMI occurs most commonly due to acute thrombosis, which, together with the rupture or erosion of plaques developed due to an atherosclerotic background, obstructs the coronary arteries secondary to local and systemic thrombogenesis.^9^ In addition, conditions that cause an imbalance in myocardial oxygen delivery and demand can also cause nonobstructive AMI.^9 , 10^ Malignant diseases are also known to cause an increase in the frequency of AMI due to vasospasm, thrombosis, accelerated atherosclerosis, and radiation-related endothelial damage.^3 , 11^ A case report stated that inferior STEMI developed in an APL patient with DIC, and acute coronary syndrome regressed dramatically quickly with heparin infusion.^12^ The authors considered that the etiology of the patient’s acute coronary syndrome was due to DIC and not to atherosclerotic plaque rupture.^12^ Cases of non thrombotic AMI secondary to hyperleukocytosis and leukostasis and thrombotic AMI secondary to DIC have been reported in the literature.^13 , 14^ Interestingly, in this case we have presented, there was no hyperleukocytosis, leukostasis or DIC. It is also known that especially in APL, leukemic promyelocytes can increase the expression of tissue factor (TF) and cancer procoagulant (CP), thus leading to thrombosis.^7^ The development of existing thrombosis without coagulopathy can be thought to be secondary to the procoagulant content in the granules of abnormal promyelocytes.

Co-occurrence of cerebrovascular diseases (CVD) with leukemia, which have risk factors similar to AMI, is uncommon in the community. However, a study has shown that the risk of cerebrovascular disease development is increased in leukemia patients compared to the normal population. The mortality rate increases five times higher in leukemia patients with CVD development compared to leukemia patients without it.^15 , 16^ Ischemic CVDs are an extremely rare complication in leucemia.^15^ Besides, a case in the USA has been reported in which a patient presented with acute lower extremity and late coronary stent thrombosis, which was thought to be secondary to DIC, and was diagnosed with AML-M3 during hospitalization.^14^ In addition, life-threatening recurrent arterial-venous thromboembolic events (such as deep vein thrombosis, pulmonary embolism, splenic infarction, renal infarction, etc.) have been reported in APL patients in the literature, mostly during chemotherapy induction therapy.^17^

Xiao et al. reported that high WBC/D-dimer (>5) and low D-dimer/FIB (<5) could be independent predictors for thrombosis in their study on patients with APL.^18^ In the case we presented, the D-dimer/FIB ratio was low in correlation with this study (1.1), but differently, the WBC/D-dimer ratio was not high (0.2). This difference can be explained by our case being pancytopenic, while patients with very high WBC were included in the reference study.

We thought that the multiple thrombotic events experienced by the patient were primarily due to AML causing coagulopathy secondary to the procoagulant content in the granules of abnormal promyelocytes. At the same time, apical aneurysm and severe hypokinesia at the apex are also possible in the etiology of cardioembolic stroke, despite the absence of intracardiac thrombus in echocardiography after AMI.

In this case report, we present a patient with AMI who developed a *cerebrovascular accident* during follow-up and was diagnosed with AML-M3 with catastrophic results. Although AML-M3 has the best prognosis among myeloid leukemias, it is obvious that the prognosis is adversely affected by AMI and cerebrovascular accident coexistence. Although DIC and leukostasis are blamed in most AML-related AMI cases reported in the literature, these clinical pictures were not present in our case. APL should be considered in the differential diagnosis of pancytopenia accompanying thrombosis.

In cases that cause multivessel ischemia, hematological diseases should be included in the etiological investigation. Possible pathologies should be evaluated by studying complete blood count, D-dimer, and FIB. Diagnosing the underlying primary disease is important in determining the treatment method (such as antithrombotic medication, leukapheresis, or percutaneous arterial interventions) and prognosis.
